# Tapping into Nature’s Arsenal: Harnessing the Potential of Natural Antioxidants for Human Health and Disease Prevention

**DOI:** 10.3390/foods13131999

**Published:** 2024-06-25

**Authors:** Víctor Pinilla-González, Catalina Rojas-Solé, Francisca Gómez-Hevia, Tommy González-Fernández, Antonia Cereceda-Cornejo, Silvia Chichiarelli, Luciano Saso, Ramón Rodrigo

**Affiliations:** 1 Molecular and Clinical Pharmacology Program, Institute of Biomedical Sciences, Faculty of Medicine, University of Chile, Santiago 8380000, Chile; victorpinilla@ug.uchile.cl (V.P.-G.); catalinarojass@ug.uchile.cl (C.R.-S.); mgomezh@ug.uchile.cl (F.G.-H.); tommy.gonzalez@ug.uchile.cl (T.G.-F.); antoniacereceda@ug.uchile.cl (A.C.-C.); 2 Department of Biochemical Sciences “A. Rossi-Fanelli”, Sapienza University of Rome, 00185 Rome, Italy; silvia.chichiarelli@uniroma1.it; 3 Department of Physiology and Pharmacology “Vittorio Erspamer”, Faculty of Pharmacy and Medicine Sapienza University, P.le Aldo Moro 5, 00185 Rome, Italy; luciano.saso@uniroma1.it

**Keywords:** natural antioxidants, protective mechanisms, oxidative stress, ischemia–reperfusion injury, non-alcoholic fatty liver disease, polyphenols, vitamins

## Abstract

Numerous natural antioxidants commonly found in our daily diet have demonstrated significant benefits for human health and various diseases by counteracting the impact of reactive oxygen and nitrogen species. Their chemical properties enable a range of biological actions, including antihypertensive, antimicrobial, anti-inflammatory, anti-fibrotic, and anticancer effects. Despite promising outcomes from preclinical studies, ongoing debate persists regarding their reproducibility in human clinical models. This controversy largely stems from a lack of understanding of the pharmacokinetic properties of these compounds, coupled with the predominant focus on monotherapies in research, neglecting potential synergistic effects arising from combining different antioxidants. This study aims to provide an updated overview of natural antioxidants, operating under the hypothesis that a multitherapeutic approach surpasses monotherapy in efficacy. Additionally, this study underscores the importance of integrating these antioxidants into the daily diet, as they have the potential to prevent the onset and progression of various diseases. To reinforce this perspective, clinical findings pertaining to the treatment and prevention of non-alcoholic fatty liver disease and conditions associated with ischemia and reperfusion phenomena, including myocardial infarction, postoperative atrial fibrillation, and stroke, are presented as key references.

## 1. Introduction

Nature offers a rich array of antioxidant compounds, including polyphenols, carotenoids, and vitamins, many of which are abundant in our daily diets. These natural products serve as vital reservoirs of therapeutic agents, deeply ingrained in both traditional and modern medicine due to their ability to combat diseases and promote overall well-being. Recognized for their importance, they are invaluable resources for developing innovative medications and addressing various health challenges.

Numerous physiological, biochemical, and molecular processes underlie the action of natural products in promoting health or contributing to pathological conditions, as demonstrated by both in vitro and in vivo studies. These products notably modulate pathways associated with oxidative stress, inflammation, and apoptosis, which are inherently interconnected [1].

Oxidative stress denotes an imbalance between the production of reactive oxygen species (ROS) and the body’s antioxidant defense mechanisms, leading to cellular dysfunction and damage. In contrast, inflammation represents an immune system response to harmful stimuli, characterized by the mobilization of immune cells and the release of inflammatory mediators [1]. Furthermore, inflammation can exacerbate oxidative stress, and both oxidative stress and inflammation can activate pro-apoptotic pathways, resulting in cell death. Apoptosis, or programmed cell death, is a crucial mechanism for maintaining tissue homeostasis and eliminating damaged or abnormal cells [1]. The disruption of these processes can contribute to the onset and progression of various diseases, including metabolic disorders, immune-related diseases, and cancers. Therefore, understanding the mechanisms underlying these processes and identifying potential therapeutic targets for their regulation is crucial for the prevention and treatment of these diseases [1].

In the realm of nutritional and medicinal research, natural medicinal foods have emerged as a subject of significant interest. These foods, which blend nutritional and medicinal properties, offer extracts with unique qualities that contribute to disease prevention and treatment, along with a myriad of health benefits, including polyphenols, flavonoids, terpenes, and alkaloids [1]. Indeed, there has been a noticeable trend towards the preference for natural food ingredients in recent years, owing to their perceived safety and widespread availability [2]. Researchers are increasingly exploring how these natural foods can complement conventional treatments, improve overall health outcomes, and contribute to the development of functional foods and nutraceuticals [3]. However, despite promising findings from preclinical studies, there remains a lack of adequate replication of these results in human clinical models, leading to ongoing debate regarding the suitability of their clinical application.

Against this background, one of the objectives of the present study is to provide an updated overview of data demonstrating the beneficial effects and limitations of studies involving natural antioxidants. These compounds, found in various diets around the world, have established themselves as fundamental components of a balanced diet. Additionally, we aim to explore the hypothesis that a combination of these compounds, as part of a multi-therapy approach, may yield greater benefits when combined based on their physicochemical properties and mechanisms of antioxidant action. This strategy could mitigate the adverse effects associated with conventional therapies, thereby reducing patient morbidity and mortality.

Supporting this perspective, this study presents findings from clinical models of specific human diseases mediated by oxidative stress. Conditions such as non-alcoholic fatty liver disease and ischemia–reperfusion injury are prevalent worldwide and impose a substantial economic burden, particularly given the aging population [4,5]. These pathologies have been focal points for exploring synergistic approaches with natural antioxidants, leading to the development of therapies that have shown promising results in both preclinical and clinical trials [6,7]. These findings serve to underscore the potential of natural antioxidants as integral components of dietary interventions aimed at promoting overall health and combating disease.

## 2. Oxidative Stress and Antioxidant Defense System

Oxidative stress has been identified as a deterioration process involved in various pathological conditions, characterized by an imbalance between the generation of prooxidant substances and the antioxidant potential. This imbalance leads to alterations in redox homeostasis and damage to macromolecules [8]. Among the most relevant pro-oxidant substances are free radicals, which, in adequate concentrations, play vital roles in the cell and the organism, such as defense against microorganisms, the activation of transcription factors, and protein phosphorylation, among others. However, when produced in excess, these radicals can cause damage, as they are highly reactive molecules due to the presence of unpaired electrons in their outer orbit, allowing them to interact with other molecules and modify their configuration and function, as occurs with biomolecules [9,10].

The main free radicals include reactive oxygen species (ROS) and reactive nitrogen species (RNS), which can sometimes combine to form even more reactive species known as reactive oxygen and nitrogen species (RONS) [11]. The generation of free radicals in cells occurs due to the action of different enzymes and cellular processes. For example, among the most important sources of free radicals is oxidative phosphorylation in the inner membrane of mitochondria (OXPHOS), NADPH oxidase (NOX) activity present in activated leukocytes during the respiratory burst process, as well as the action of other enzymes such as myeloperoxidase (MPO) and uncoupled nitric oxide synthase (NOS) [12,13]. Additionally, free radicals can be generated spontaneously, as in the presence of transition metals. For example, iron in its free form reacts with hydrogen peroxide (H2O2) to form hydroxyl radicals [14].

**Figure 1:**
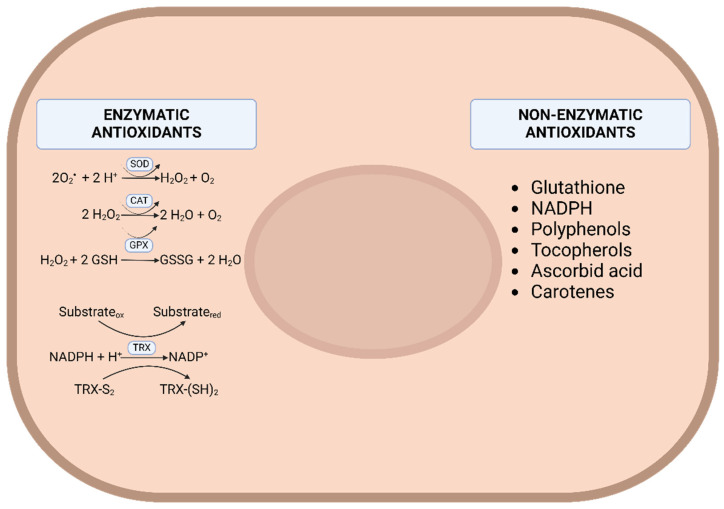
Basic cellular mechanisms of defense against oxidative stress. SOD, superoxide dismutase. CAT, catalase. GPX, glutathione peroxidase. TRX, thioredoxin.

Since an excess of free radicals can cause damage, cells have developed various defense mechanisms to counteract them. These mechanisms are divided into enzymatic and non-enzymatic antioxidants. The latter can, in turn, be categorized based on whether they are synthesized naturally in the body or must be ingested through the diet, as is the case with the natural antioxidants mentioned in the previous section. Antioxidant enzymes include superoxide dismutase (SOD), catalase (CAT), glutathione peroxidase (GPX), thioredoxin (TRx), and others [12] (Figure 1). The expression of these enzymes is largely controlled by the nuclear erythroid 2-related factor (Nrf2)/Keap1 system, which constitutes one of the most studied adaptive response systems to oxidative stress [15]. 

## 3. Natural Antioxidant

In this section, we will discuss the main families of natural antioxidants present in a standard Mediterranean diet rich in fruits and vegetables, which are grouped into polyphenols, carotenoids, and vitamins. Polyphenols, abundant in fruits, vegetables, and other plant-based foods, encompass a diverse range of compounds such as flavonoids, phenolic acids, lignans, and stilbenes, each offering unique health benefits. Carotenoids, found primarily in colorful fruits and vegetables, play a crucial role in maintaining eye health and supporting immune function. Lastly, vitamins, including vitamins C and E, are essential micronutrients with powerful antioxidant properties that contribute to overall health and well-being. Together, these natural antioxidants form an integral part of a balanced diet, offering protection against oxidative stress and reducing the risk of chronic diseases.

### 3.1. Polyphenols

Polyphenols are secondary metabolites produced by various parts of plants, including fruits, flowers, leaves, and barks [16]. Widely distributed in plant-based foods, they contribute to their color, flavor, and pharmacological activities [2].

Polyphenols are classified based on their chemical structure, characterized by one or more phenolic rings in their molecular composition [17]. They are broadly categorized into four main groups: flavonoids, phenolic acids, lignans, and stilbenes. Flavonoids, the most abundant class, can be further subdivided into subgroups such as anthocyanins, flavanols, flavanones, flavones, flavonols, and isoflavones. Phenolic acids are the second most common, followed by lignans and stilbenes [17].

The specific polyphenol content of foods varies widely depending on factors such as plant variety, ripeness, processing methods, and cooking techniques [18]. During digestion, some polyphenols, like anthocyanins, are absorbed in the small intestine, while others, like flavonoids, require hydroxylation by digestive enzymes before absorption by epithelial cells [2].

Polyphenols possess antioxidant properties and can modulate the expression of various proinflammatory genes, contributing to the regulation of inflammatory signaling. They exert anti-inflammatory effects through radical scavenging, metal chelation, NOX inhibition, the modulation of the mitochondrial respiratory chain, the inhibition of enzymes involved in ROS production (e.g., xanthine oxidase), and the augmentation of endogenous antioxidant enzymes [2].

This section will delve deeper into the actions of three specific polyphenols: resveratrol, curcumin, and quercetin. Extensively researched for their potential health benefits, these compounds offer insight into how polyphenols influence human physiology and contribute to disease prevention. We will explore their roles in modulating inflammatory pathways, protecting against oxidative stress, and other mechanisms contributing to their beneficial effects on health. Additionally, we will examine the emerging significance of the phenolic acid ferulic acid, known for its potent antioxidant properties and its ability to modulate various biological pathways, thereby enhancing its potential therapeutic applications.

#### 3.1.1. Resveratrol

Resveratrol, known chemically as 3,5,4′-trans-trihydroxystilbene, belongs to the stilbene family and is primarily found in grapes, red wine, and various plant-based foods like peanuts, berries, and tea. Produced by over 70 plant species in response to infection, stress, injury, bacteria, fungal infections, and UV radiation [19], resveratrol has shown a wide range of bioactivities in vitro studies. These include antioxidant, anti-inflammatory, immunomodulatory, hypotensive, and hypolipidemic properties, demonstrating efficacy in preventing and treating diseases such as cancer, cardiovascular disease, neurodegenerative diseases, and obesity [20]. In this context, the so-called “French paradox,” which refers to the low incidence of cardiovascular disease among the French despite a diet rich in saturated fats, especially in the form of cheese and butter, makes sense. However, the consumption of red wine, which is characterized by being rich in polyphenols such as resveratrol, is also high [21].

The molecular mechanisms underlying resveratrol’s biological function include its ability to actively scavenge free radicals, chelate metals, and modulate signaling pathways associated with phosphoprotein kinase B (Akt) and phosphoprotein kinase C, as well as activating AMPK and NRF-2 [19]. However, while these observations stem mainly from in vitro studies, in vivo research has struggled to effectively demonstrate the major benefits of resveratrol. This is largely due to its limited oral bioavailability, influenced by hepatic metabolism and variable absorption [22]. Moreover, the presence of hydroxyl groups in resveratrol allows it to associate with carbohydrates and proteins in the diet, reducing its absorption [22]. Furthermore, there is significant interindividual variability in polyphenol metabolism. Various studies have shown that factors such as age, sex, genotype, and, most importantly, gut microbiota, play a crucial role in determining these differences [23,24]. These factors lead to variations among individuals in the metabolism and bioavailability of resveratrol, resulting in a heterogeneous response to its consumption. This variability represents a key aspect that must be considered in research and therapeutic applications.

Regarding its safety profile, resveratrol has been associated with various adverse effects, which depend on the type of clinical study. Common side effects include dizziness and headache, with less frequent occurrences of epididymitis. However, safety has been reported across varying doses of the compound (e.g., 0.5, 1.0, 2.5, and 5.0 g) [25]. Higher doses, such as 2.5 and 5.0 g, have been linked to side effects like diarrhea, nausea, and abdominal discomfort [25]. Despite these adverse effects, resveratrol’s side effects are generally considered mild compared to its potential health benefits in various pathologies.

#### 3.1.2. Quercetin

Quercetin, with a chemical structure of 2-(3,4-dihydroxyphenyl)-3,5,7-trihydroxy-4H-1-benzopyran-4-one, stands out as a prominent member of the non-toxic natural flavonoid family. It is abundantly present in fruits such as grapes and peaches, as well as in vegetables like onions and garlic [26,27]. Moreover, owing to its lipophilic nature, quercetin easily crosses the intestinal membrane through simple diffusion [26]. Additionally, quercetin is often found alongside other phytochemicals, vitamins, and minerals in whole foods, leading to potential synergistic effects that enhance their individual benefits. For instance, consuming quercetin-rich foods with those containing vitamin C may boost quercetin absorption due to their possible synergistic interaction [28,29].

Quercetin possesses remarkable anti-aging properties attributed to its antioxidant, anti-apoptotic, and anti-inflammatory attributes. It actively participates in enhancing mitochondrial function and exhibits iron-chelating abilities, rendering it effective against a process known as ferroptosis [2]. However, it is important to note that the clinical application of quercetin faces limitations due to its low bioavailability and solubility [26,30].

Numerous research studies have unequivocally demonstrated that quercetin treatment effectively suppresses ROS generation and ameliorates mitochondrial dysfunction, thereby contributing to the preservation of normal mitochondrial balance and function [26]. These effects are closely linked to changes in the electron cloud within the aromatic ring. When quercetin interacts with a free radical, it either donates electrons or provides hydrogen, resulting in the formation of a new stable group through the orchestrated spin action of the aromatic nucleus. Consequently, this interrupts or delays the oxidation reaction of the substance, highlighting the positive correlation between quercetin’s antioxidant capacities and the stability of the formed group [27].

**Figure 2:**
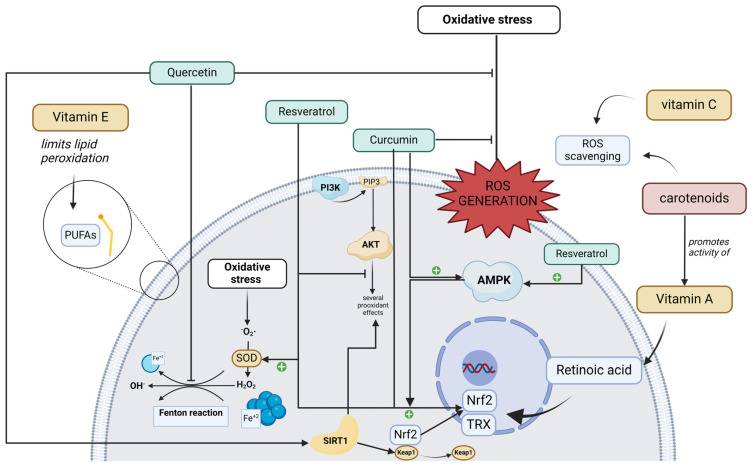
Inter-related molecular mechanisms through which natural antioxidants exert their antioxidant effects. AKT, protein kinase B; AMPK, AMP-activated protein kinase; Fe^2+^, ferrous iron; Fe^3+^, ferric iron; Keap1, Kelch-like ECH associated protein 1; Nrf-2, nuclear factor-erythroid 2-related factor 2; PI3K, phosphoinositide 3-kinase; PIP3, phosphatidylinositol (3,4,5)-triphosphate; PUFAs, polyunsaturated fats; ROS, reactive oxygen species; SIRT-1, sirtuin 1; SOD, superoxide dismutase; TRX, thioredoxin.

Furthermore, quercetin has been shown to exert neuroprotective effects against chronic aging-related diseases. This is achieved by targeting the SIRT1 pathway, which regulates cellular senescence and several aging-related cellular processes, including SIRT1/Keap1/Nrf2/HO-1- and PI3K/Akt/GSK-3b-mediated oxidative stress, SIRT1/NF-κB-mediated inflammatory responses, SIRT1/PGC1alpha/eIF2alpha/ATF4/CHOP-mediated mitochondrial damage, and SIRT1/FoxO-mediated autophagy (Figure 2) [26].

Like other polyphenols, quercetin faces significant limitations for pharmaceutical use due to its poor solubility and low bioavailability. Studies indicate that the total bioavailability of quercetin is merely 2% following oral administration [31]. To overcome these barriers, the pharmaceutical industry has advanced the development of nanoformulations. These nanoformulations have been shown to enhance the bioavailability and stability of quercetin, as well as improve its antioxidant capacity. Consequently, they have recently been widely utilized as complementary therapies in the treatment of various cancers [32].

Regarding the safety of quercetin in humans, results from a phase I clinical trial by Ferry et al. (1996), in which different doses of the flavonoid were administered intravenously, are currently available [33]. The results showed that patients who received doses up to 10.8 mg/kg experienced no adverse effects, while those treated with a higher dose (51.3 mg/kg) suffered from injection site pain, emesis, dyspnea, and nephrotoxicity. However, these results cannot be directly compared to those obtained from oral administration, where few studies have reported adverse effects. For instance, quercetin supplementation (1000 mg/day for 1 month) reported mild adverse effects such as nausea, headache, and tingling in the extremities [34]. Similarly, in vitro studies have suggested that potential safety issues may arise if mega doses of flavonoids are consumed daily [35]. However, the exact threshold for what constitutes a megadose of quercetin has not been defined. Therefore, until a tolerable upper limit is established, the consumption of large amounts of flavonoids in concentrated supplement form cannot be considered safe, and the safety potential of higher doses needs to be assessed in vivo.

#### 3.1.3. Curcumin

Turmeric (*Curcuma longa*), a traditional Chinese spice and medicinal herb, holds a rich history of application in managing various health conditions, particularly those associated with inflammation [36,37]. Turmeric comprises a trio of curcuminoids (curcumin, demethoxycurcumin, and bisdemethoxycurcumin), alongside volatile oils (natlantone, tumerone, and zingiberone), proteins, sugars, and resins [36]. A key component of this versatile herb is curcumin, a bioactive compound chemically known as (1E-6E)-1,7-bis(4-hydroxy-3-methoxyphenyl)-1,6-heptadiene-3,5-dione. Curcumin emerges as a remarkable molecule with far-reaching impact, targeting numerous molecular pathways associated with inflammatory processes. It exhibits a multifaceted character, offering antioxidative, anti-inflammatory, hepatoprotective, anticancer, and other valuable properties [37].

Curcumin holds significant promise as a pharmacological agent due to its pivotal roles in addressing oxidative stress, managing inflammatory responses, and influencing apoptosis. It demonstrates favorable outcomes in conditions encompassing metabolic disorders, immune-related ailments, and various cancers [1]. Notably, curcumin showcases its ability to scavenge ROS, diminish the production of proinflammatory cytokines, and modulate diverse signaling pathways associated with apoptosis. Even at high doses, curcumin maintains a favorable safety profile with no significant adverse effects reported [1]. Moreover, pre-treatment with curcumin stands out for its capacity to regulate the expression of antioxidant enzymes via the nuclear factor erythroid 2-related factor 2 (Nrf2) signaling pathways, thereby stabilizing ROS levels (Figure 2). The transcription factor Nrf2 plays a pivotal role in the cellular response to oxidative stress by regulating the expression of genes encoding antioxidant enzymes and detoxifying proteins. Recent research indicates that curcumin can alleviate intestinal barrier injury and mitigate mitochondrial damage induced by oxidative stress through the activation of the AMP-activated protein kinase (AMPK) pathway [1].

Regarding its role in managing inflammatory responses, curcumin’s effectiveness lies in its ability to obstruct the nuclear factor kappa-light-chain-enhancer of activated B cells (NF-κB) pathway—a crucial element in the oxidative and inflammatory process [1]. Furthermore, it has been observed that curcumin exerts influence over multiple apoptotic pathways, including the death receptor and endoplasmic reticulum stress-induced apoptosis pathways [1].

Expounding on its pharmacological attributes, curcumin is a powerhouse, endowed with properties spanning antioxidant, anti-inflammatory, hepatoprotective, anticancer, antidiabetic, cardiovascular protective, neuroprotective, immune regulatory, metabolic syndrome protective, and even eye-protective effects. It serves as a versatile solution applied in the management of various conditions, including intervertebral disk issues, herniation, cancer-related pain, arthritis, delayed-onset muscle soreness, burn pain, mental stress, hypochondriac pain, mania, visceral pain, and musculoskeletal pain. In essence, curcumin’s multifaceted benefits encompass its antioxidant potential through AMPK/Nrf2/ARE/Keap1 pathway activation, its anti-inflammatory attributes via NF-κB/AP-1/MAPK pathway inhibition, and its ability to counteract apoptosis through the suppression of JAK/STAT and ER stress-induced pathways, while simultaneously activating PI3K/AKT/mTOR pathways, especially in noncancerous ailments [1]

The safety profile of curcumin is generally acceptable, as it has not been shown to cause serious adverse effects. Studies with oral curcumin formulations indicate that doses between 1 and 4 g/day are well tolerated, with the most common side effects being gastrointestinal discomfort, such as nausea, vomiting, or diarrhea [38,39,40]. However, a challenge with oral formulations is their low bioavailability. To address this, liposomal formulations for intravenous administration have been developed, which have also been found to be safe up to doses of 120 mg/m^2^, with changes in red blood cell morphology being a sign of dose-limiting toxicity [41].

Despite its acceptable safety profile, curcumin has been shown to interact with enzymes that metabolize other drugs [42]. Therefore, it should be used with caution when combined with antidepressants, antibiotics, cardiovascular drugs, anticoagulants, or chemotherapeutics.

#### 3.1.4. Ferulic Acid

Ferulic acid (FA) is a phenolic compound with the molecular structure of 4-hydroxy-3-methoxycinnamic acid (C_10_H_10_O_4_), which is part of the plant cell wall and is widely distributed in nature. However, its main food sources are legumes and cereals. This compound has been shown to possess various pharmacological properties, including antioxidant and anti-inflammatory effects, as well as beneficial effects on parameters related to diabetes and hyperlipidemia [43,44]. 

In terms of its antioxidant mechanism of action, FA inhibits ROS production and aldose reductase activity, while simultaneously activating the PI3K/Akt signaling pathway to perform its antioxidant function [40]. Furthermore, activation of the Nrf2/HO-1 pathway enhances the antioxidant effect of FA by promoting the nuclear translocation of HO-1 [40]. At the endothelial level, FA negatively regulates the expression of NO/ET-1 and factors related to vascular endothelial function, such as VEGF, PDGF, and HIF-1alpha, thereby protecting endothelial cells and enhancing angiogenesis, which is crucial for maintaining normal vascular endothelial function [40]. Additionally, FA interferes with the proinflammatory activity of macrophages, reducing the secretion of cytokines such as TNF-alpha, IL-6, and IL-1β [44,45].

The safety profile of ferulic acid is favorable. For example, daily supplementation of 1 g/day for 6 weeks has not been associated with any adverse effects, indicating that this dose appears to be safe [46]. Other formulations, such as sodium ferulate for intravenous administration, have also been found to be safe. Clinical trials evaluating the efficacy of sodium ferulate have reported only minor adverse events, which were related to the infusion rate and allergic reactions [47].

### 3.2. Carotenoids

Carotenoids constitute a group of natural pigments abundant in many fruits, vegetables, and plants. These fat-soluble compounds are responsible for the vibrant red, orange, and yellow hues observed in various foods, including fruits, vegetables, and fish [48]. Upon ingestion, they are released from the food matrix and absorbed in the intestine, incorporating into micelles that diffuse into the plasma membrane of enterocytes. Subsequently, they are transported into circulation via high-density lipoproteins (HDLs) and low-density lipoproteins (LDLs) [49]. Depending on their chemical structure, carotenoids can be categorized into carotenes and xanthophylls. Carotenes, such as alpha-carotene, beta-carotene, and lycopene, are non-oxygenated, whereas xanthophylls, such as zeaxanthin, astaxanthin, and canthaxanthin, are oxygenated derivatives [50].

Carotenoids exhibit various biological activities, including provitamin A activity, immune response stimulation, the modulation of gap junction communication, and the regulation of cell cycle apoptosis [50]. Their primary antioxidant activity arises from their capacity to scavenge reactive oxygen species (ROS) through processes like electron transfer (oxidation and reduction), hydrogen abstraction, and addition reactions [48]. Carotenoids are further classified into three classes based on their antioxidant capacity: the first class demonstrates minimal antioxidant activity; the second class, comprising beta-carotene and lycopene, shows significant antioxidant activity but may also exhibit pro-oxidant properties; and the third class, represented by astaxanthin, exhibits robust antioxidant capacity devoid of any pro-oxidant nature [51].

Astaxanthin (AX), a xanthophyll, has garnered significant attention in recent years. This orange-red compound is abundant in many aquatic animals, such as salmon and shrimp, primarily synthesized by the microalgae consumed by these fish, where it accumulates. Among microalgae, Haematococcus pluvialis stands out for its remarkable ability to concentrate astaxanthin [52]. AX possesses the capacity to neutralize various reactive oxygen species (ROS) and nitrogen species (NOS), with its exceptionally potent superoxide anion scavenging activity being particularly noteworthy. Additionally, owing to its lipophilic nature, it can inhibit lipid peroxidation, thereby safeguarding mitochondrial membranes against oxidative damage [53,54]. The beneficial effects of AX encompass protection against UV damage, anti-inflammatory and immunomodulatory activities, cardioprotective effects, and anticancer properties, among others [53]. Regarding the safety of its use, several studies report an excellent safety profile for short-term daily doses of 100 mg and long-term doses of 8–12 mg [55,56,57,58]. Only a few mild adverse effects have been reported, such as a change in stool color or an increase in the number of stools.

Another natural compound found abundantly in many red fruits and vegetables is lycopene. Studies have shown that it reaches its highest concentrations in tomato powder and sun-dried tomatoes compared to fresh tomatoes or other forms of this fruit [59]. Lycopene exhibits antioxidant activity, playing a role in conditions such as cardiovascular diseases and cancer. It functions as a scavenger for singlet oxygen and peroxyl radicals, while also targeting other free radicals like hydrogen peroxide, nitrogen dioxide, and hydroxyl radicals. Additionally, lycopene is believed to enhance the cellular antioxidant defense system by regenerating non-enzymatic antioxidants such as vitamins C and E [59,60].

### 3.3. Vitamins

#### 3.3.1. Vitamin C

Ascorbic acid (L-ascorbic acid or L-ascorbate), commonly known as vitamin C, is a water-soluble vitamin essential for the proper functioning of the human body. In most mammals, it can be produced in a multi-step pathway from glucose, but humans are the exception, as they lack the enzyme L-gulonolactone oxidase, an enzyme necessary for the synthesis of ascorbic acid, so it must be ingested in the diet [61,62]. Deficiency of this vitamin has been associated with the development of scurvy disease, which manifests itself as general weakness, fatigue, myalgia, arthralgia, lack of appetite, decreased immunity, tendency to bruise, and inflammation of the gums and bleeding, demonstrating the diversity of processes in which vitamin C is involved [63]. The main sources of vitamin C in the diet are fruit and vegetables, as animal products have a relatively low vitamin C content, with the exception of cattle liver and some fish eggs. Among the foods richest in ascorbic acid in fruits are carambola, guava, blackcurrant, kiwi, and strawberries, while among vegetables, broccoli, cabbage, and peppers stand out [64,65].

The biological functions of vitamin C are the result of its ability to act as a reducing agent. In this way, vitamin C is capable of reducing oxidizing agents by donating electrons, which gives it its name as an antioxidant, but this does not always occur, given that when reducing elements such as transition metals like copper or iron, they are capable of generating reactive oxidizing species such as superoxide anion and hydrogen peroxide through the Fenton reaction [66,67]. When ascorbic acid is oxidized, it gives rise to dehydroascorbic acid (DHA), which is a more stable species and has a certain affinity for glucose transporters, through which, it can be taken up and reduced to ascorbic acid inside the cell or metabolize to 2,3-dichetogulonic acid to form different metabolites, including oxalate [61,68,69]. In this context, some functions related to its ability to donate electrons include its participation as an enzymatic cofactor, participating in various reactions such as the hydroxylation of procollagen or HIF-1, histone demethylation, and norepinephrine synthesis, among others [70,71]. In terms of its role as an antioxidant, vitamin C acts directly as a potent scavenger of reactive oxygen species (ROS) and in parallel generates indirect effects on the redox state, by recycling other antioxidant molecules such as vitamin E or reducing Nf κB levels or preventing BH4 oxidation [72]. It is precisely this great versatility of vitamin C that has motivated the development of different studies aimed at better understanding its physiology, as well as its participation in different pathophysiological processes, in order to implement its clinical use in the treatment of different diseases.

The safety of ascorbic acid has been studied in various settings for the treatment of different pathologies, and most studies have shown it to have a good safety profile. A Cochrane review investigating the use of vitamin C for the prevention and treatment of the common cold concluded that ascorbic acid is safe when supplemented at doses between 0.25 and 2 g/day [73]. In more extreme cases, a review by Böttger et al. examined the efficacy and safety of high-dose vitamin C treatment (up to 3 g/kg) administered intravenously as monotherapy for various types of malignancies [74]. The results indicated that this treatment is safe and has no significant toxicity. The most common adverse effects observed with the use of high-dose vitamin C include hypokalemia, hypernatremia, hypertension, and anemia [74]. However, the most serious events, such as pulmonary embolism and pneumonia, were associated with the underlying disease rather than the treatment itself [74].

#### 3.3.2. Vitamin A

Vitamin A is an essential micronutrient for our body, which is associated with the proper functioning of our body, participating in various processes such as reproduction, embryogenesis, vision, cell growth and differentiation, and immune function, among others [75,76,77]. Thus, vitamin A deficiency is associated with the development of night blindness, xerophthalmia, xeroderma, and frequent infections, among other disorders [75,78]. The term vitamin A includes a large number of fat-soluble compounds grouped under the term retinoids. These compounds can be divided into two groups depending on whether they are of animal or vegetable origin. The group of animal origin is mainly composed of retinol, also known as preformed vitamin A, since it is a substance that acts as a precursor of the most active form of vitamin A, which is retinoic acid [79]. On the other hand, plant-derived retinoids are categorized as provitamin A compounds, which include some carotenoids that can be metabolized to retinol in our body, including alpha-carotene, beta-carotene, and beta-cryptoxanthin, since they are the only ones found in significant quantities in the human diet [80,81].

When it comes to dietary sources of vitamin A, preformed vitamin A in the form of retinol found in animal-based foods plays a significant role. Noteworthy sources include meat, milk, eggs, and fish [82]. Regarding the consumption of carotenes, as it was mentioned previously, products such as red and orange fruits and vegetables are rich in beta-carotene and beta-cryptoxanthin; thus, some foods rich in provitamin A are carrots, tomatoes, tangerines, persimmons, red peppers, papaya, mango, and loquat, among others [83,84].

Vitamin A is mainly obtained orally through the diet, but it can also be used as a drug, with different routes of administration such as intramuscular or topical use. In order for retinol to be used by target cells, it must be oxidized to retinoic acid, which corresponds to the active form of vitamin A [85].

As for the antioxidant function of vitamin A, it is a controversial function, given that it does not exercise its function directly as vitamin C or carotenoids do, nor is its antioxidant role so clear, given that at certain concentrations, it can act as a pro-oxidant [86]. In this sense, it is postulated that its function in the redox balance would be mediated mainly by the transcriptional regulator retinoic acid, which has the ability to exert genomic actions that affect the antioxidant response. In this context, for example, a study with human airway epithelial cells showed that the antioxidant enzyme thioredoxin (TRX) gene contains several elements sensitive to retinoic acid (RARE), which would indicate that retinoic acid, together with its receptor, interacts with the gene, participating in the transcription of the thioredoxin enzyme at least in the respiratory epithelium [87]. In addition, studies have linked retinoic acid to the modulation of Nrf2 signaling (Figure 2), which is a transcription factor that promotes the expression of antioxidant enzymes in response to increased oxidative stress. One study with rat intestinal cells found that retinoic acid decreased Nrf2-driven gene expression [88], but another study reported that at pharmacological doses, retinoic acid in mice and cultured cells increased the expression of Nrf2-responsive genes, contradicting the previous study [89]. In addition to these studies, there is the finding reported by researchers studying diabetic nephropathy demonstrating that rats treated with retinoic acid attenuated ROS production and lipid peroxidation and, in parallel, increased Nrf2 levels [90]. This suggests that the action of vitamin A as an antioxidant is unclear and that its function should not be confused with carotenoids, whose antioxidant function, as seen previously, is better documented.

Research on the safety profile of vitamin A has been challenging due to the potential adverse health effects associated with both hypovitaminosis and hypervitaminosis A, and thus, the available evidence is limited. One study in non-pregnant women found that oral supplementation with vitamin A at various doses (4000, 10,000, and 30,000 IU) over a three-day period maintained plasma concentrations within or slightly above the physiological range, suggesting that these doses were not teratogenic [91]. Additionally, studies have been conducted with trans-retinoic acid, a natural derivative of vitamin A, which has been used as an adjunctive therapy to chemotherapy in certain malignancies. For example, the recommended dose of trans-retinoic acid for the treatment of acute promyelocytic leukemia is 45 mg/m^2^ daily for 15 days, and this regimen has shown an acceptable toxicity and side-effect profile [92].

#### 3.3.3. Vitamin E

Vitamin E is a micronutrient which, like vitamin A, is a fat-soluble vitamin. The term vitamin E groups eight different compounds, corresponding to alpha-, beta-, gamma-, and delta-tocotrienol and alpha-, beta-, and gamma-tocopherol [93]. These compounds are only synthesized by organisms performing photosynthesis, so we must acquire them through our diet [94]. Vitamin E deficiency is rare, as it is unlikely to be due to a diet low in vitamin E, but it is rather related to diseases affecting lipid absorption. Symptoms of deficiency include ataxia, peripheral neuropathy, skeletal myopathy, retinopathy, and impaired immune response, among others [95]. The main sources of vitamin E are nuts such as peanuts or walnuts, seeds, and vegetable oils. Among vegetable oils, wheat germ oil, olive oil, and sunflower oil have the highest proportion of alpha-tocopherol compared to other vegetable oils, such as sesame or soybean oil, where gamma-tocopherol has the highest concentration [96,97].

At the physiological level, it is recognized that it is primarily alpha-tocopherol that meets most of the body’s vitamin E requirements, as it is the form that the liver is able to secrete via the alpha-tocopherol hepatic transfer protein (alpha-TTP) [98]. The other vitamin E congeners are postulated to be metabolized to water-soluble carboxyethylhydroxychromanol (CEHC) compounds and eliminated via bile and urine [99]. Among the dietary factors affecting vitamin E absorption, alpha-tocopherol has been shown to compete with the absorption of cholesterol, gamma-tocopherol, carotenoids, and the other fat-soluble vitamins (A, D, and K) [100,101].

As a fat-soluble vitamin, vitamin E is usually deposited in the plasma membranes of various cells, and it is at this site that it performs two main functions, acting as a membrane stabilizer and as an antioxidant [102]. It exerts its antioxidant function by limiting the lipid peroxidation of polyunsaturated fatty acids (PUFAs) (Figure 2), intervening directly in propagation reactions by scavenging lipid peroxyl radicals [103]. This reaction involves alpha-tocopherol being oxidized to form the alpha-tocopheroxyl radical, which is significantly more stable than peroxyl radicals. This reaction is maintained by a network of antioxidants that aims to reduce the tocopheryl radical and recycle tocopherol. This network actively involves vitamin C, glutathione (GSH), and NADPH as substrates and glutathione peroxidase (GPx) and glutathione reductase as enzymes [104]. This ability of vitamin E to limit lipid peroxidation has led to its current position as a vitamin with the capacity to prevent ferroptosis, which is cell death involving iron-mediated phospholipid peroxidation that has been found to occur in various pathophysiological processes [105]. In addition to the antioxidant activity recently described, another field of research that has motivated the attention paid to vitamin E is its relationship with gene expression, where different studies have postulated that vitamin E indirectly modulates genes linked to the cell cycle, as well as genes involved in cholesterol and steroid metabolism, among many others [106].

Safety studies of alpha-tocopherol have shown controversial results. In 2005, Miller et al. published a meta-analysis concluding that supplementation above 400 IU increased all-cause mortality, primarily associated with increased bleeding, heart failure, hemorrhagic stroke, and an increased risk of prostate cancer [107]. However, subsequent studies have refuted this theory, as the trials produced mixed results and an overall effect could not be conclusively determined [108,109]. One mechanism associated with the adverse effects of long-term alpha-tocopherol use is its interaction with vitamin K. Excessive supplementation has been proposed to lead to vitamin K depletion in tissues, potentially promoting alterations in blood clotting, vascular calcification, and cancer prevention [110]. Although the exact mechanism by which excess alpha-tocopherol interferes with vitamin K levels is unknown, a prudent recommendation for users of vitamin E supplements is to consume a diet rich in green leafy vegetables to ensure adequate vitamin K intake.

## 4. Diseases Associated with Oxidative Stress in Humans

In this section, we will provide a concise overview of the role of oxidative stress in various pathologies, aiming to elucidate potential therapeutic targets that could be effectively addressed by natural antioxidants.

### 4.1. Non-Alcoholic Fatty Liver Disease

Non-alcoholic fatty liver disease (NAFLD) is characterized by the accumulation of lipids in the liver. This condition spans a spectrum from benign forms, such as non-alcoholic fatty liver disease, to non-alcoholic steatohepatitis, which can progress to fibrosis and cirrhosis. Worldwide, the prevalence of NAFLD is around 25%, with a marked increase in Western countries and becoming more frequent in patients suffering from chronic non-communicable diseases, such as obesity, type 2 diabetes mellitus, dyslipidemia, and metabolic syndrome, and it is expected to be the main indication for liver transplantation within the next 10 years [111,112].

Regarding its pathogenesis, although the mechanisms driving the development of NAFLD are not yet fully understood, two crucial stages in the progression of the disease have been identified. The first is related to lipid accumulation in hepatocytes, leading to insulin resistance. On the other hand, the second stage involves molecular changes closely linked to oxidative stress. In this context, it has been observed that lipid peroxide levels are markedly higher in patients with steatosis and metabolic syndrome compared to healthy patients. In addition, reduced levels of antioxidants such as glutathione, superoxide dismutase, and catalase have been observed [111,112].

In this context, oxidative stress, resulting from ROS generation, is considered a determinant factor in the progression to non-alcoholic steatohepatitis (NASH). In addition, the overproduction of ROS promotes lipid peroxidation, resulting in the formation of aldehyde products and increased levels of various cytokines (such as TNF-alpha, TGF-β, Fas ligand, and IL-8). Likewise, increased TNF-alpha signaling causes the activation of Jun-N-terminal kinase and other oxidative stress-sensitive transcription factors, such as NF-κB, thus generating a vicious cycle, as this amplifies the production of inflammatory cytokines such as IL-6 and IL-1β, promoting the development of pathology [112].

### 4.2. Ischemia–Reperfusion Injury

Injury resulting from the combination of ischemia followed by reperfusion (ischemia–reperfusion injury, IRI) is a serious and pressing condition that threatens both the function and integrity of any organ or tissue. Particularly, ROS are major factors mediating this damage. 

#### 4.2.1. Coronary Artery Disease

The combination of this alteration along with recanalization therapy has emerged as a significant global health concern, marked by a high prevalence that ranks it among the primary contributors to morbidity and mortality. It is important to highlight that its incidence is constantly increasing, especially among the young population [113]. In the context of the treatment of this condition, the sudden restoration of oxygen supply to myocardial cells as a result of recanalization creates a detrimental environment for the tissue due to the formation of oxygen-generated free radicals. This leads mainly to alterations such as lipid peroxidation, especially those present in cell membranes, triggering a series of side effects associated with this therapeutic approach [114]. Along the same lines, but in the field of surgery, the injury caused by the combination of ischemia and reperfusion also poses a significant challenge, especially in procedures such as liver resections and any organ transplantation [115]. 

The pathophysiological mechanisms underlying these conditions have in common the arrest of blood flow, especially in the heart, either due to atherosclerosis or myocardial infarction, which generates a series of changes that alter various cellular functions, such as the induction of anaerobic metabolism causing a decrease in ATP and antioxidant agents, the retention of lactic acid-producing acidosis, the failure of sodium–potassium or calcium pumps causing an accumulation of sodium inside the cell and potassium outside it, and the detachment of ribosomes from the nuclear chromatin, among others. Simultaneously to the mentioned processes, the generation of ROS increases due to the decrease in antioxidant agents, causing oxidative stress and all the alterations that its appearance entails, such as endothelial dysfunction, DNA damage, and inflammation, finally resulting in cell death [116]. Undoubtedly, the pathophysiology of IRI has been shown to involve a significant perturbation in the regulation of the cellular redox state. As a result, several studies have explored various prophylaxis strategies, such as the administration of antioxidants, including vitamin C and E. However, despite the conduction of numerous clinical trials with various compounds, no particular therapy capable of reducing reperfusion injury has been identified so far [117,118].

**Figure 3:**
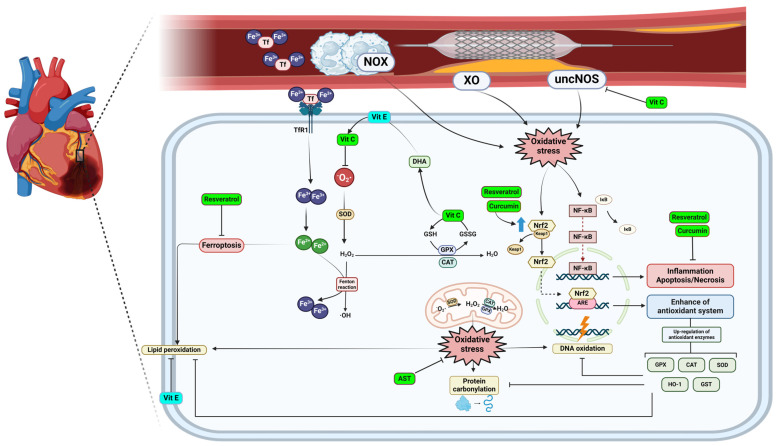
Mechanisms of action of natural antioxidants on ischemia and reperfusion injury in acute myocardial infarction. Blue upward arrows (↑) indicate upregulation. ARE, antioxidant response element; AST, astaxanthin; CAT, catalase; DHA, dehydroascorbic acid; Fe^3+^, ferric iron; Fe^2+^, ferrous iron; GPX, glutathione peroxidase; GSH, reduced glutathione; GSSG, oxidized glutathione; GST, glutathione transferase; HO-1, heme oxygenase-1; IκB, NF-κB inhibitor protein; KEAP1, Kelch-like ECH-associated protein 1; NF-κB, nuclear factor kappa-light-chain-enhancer of activated B cells; Nrf2, nuclear factor-erythroid 2-related factor 2; NOX, reduced nicotinamide adenine dinucleotide phosphate oxidase; SOD, superoxide dismutase; Tf, transferrin; TfR1, transferrin receptor protein 1; Vit C, vitamin C; Vit E, vitamin E; XO, xanthine oxidase.

There are multiple mechanisms by which reactive oxygen species (ROS) are generated. Some of these include the electron transport chain, the enzyme NADPH oxidase, the xanthine oxidase (XO) system, uncoupled nitric oxide synthase (uncNOS), and the arachidonic acid reaction catalyzed by cyclooxygenase-2, among others. Of all these mechanisms, the electron transport chain, NADPH oxidase, and xanthine oxidase are involved in postischemic oxidative stress in various organs such as the heart, liver, brain, and intestine, while NOS is particularly related to the heart, liver, and aortic endothelial cells [116]. The mechanism of production via the electron transport chain in mitochondria is predominantly the escape of electrons from complexes I, II, and III to cause the reduction of oxygen to superoxide anion, which can dismute to hydrogen peroxide [119]. On the other hand, the NADPH oxidase system consists of enzyme complexes whose sole function is the generation of reactive oxygen species (ROS). These complexes are responsible for the production of superoxide anions or hydrogen peroxide through the reduction of molecular oxygen, using NADPH as electron donor. This process occurs ubiquitously in all cells of the organism (Figure 3) [120].

#### 4.2.2. Post Operative Atrial Fibrillation 

Atrial fibrillation is the most common arrhythmia in the adult population and represents a major challenge for the medical community to treat, prevent, or cure [121]. This is largely because the underlying mechanisms leading to the onset and persistence of the arrhythmia are unknown. Acute or new-onset AF following cardiac surgery corresponds to postoperative AF (POAF), a much-feared complication, as it is associated with an increased length of hospital stay, readmission to the intensive care unit, persistent congestive heart failure, an increased risk of stroke, increased overall costs, and mortality [122,123]. This complication occurs in 20–50% of patients after cardiac surgery, with a higher prevalence in valve replacement surgery and cardiopulmonary bypass procedures [124].

The underlying pathophysiology and mechanism of POAF are multifactorial and poorly understood; however, oxidative stress and inflammation have been the main mechanisms of damage related to POAF. In this regard, surgical manipulation of the heart and pericardium, as well as the systemic injury associated with surgery, drive an immunological process characterized by both local and systemic inflammation [125,126]. To this acute inflammatory process is added the inflammatory state prior to surgery, which is mainly given by pre-existing comorbidities, such as different pathologies like hypertension, diabetes, atherosclerosis, or others, which are related to endothelial dysfunction that generates a chronic low-grade inflammatory state [127]. In this sense, the detrimental action of different inflammatory mediators on cardiac function has been well studied. For example, interleukin-6 (IL-6) induces cardiac remodeling and alters the beta-adrenergic response of the heart, while interleukin-8 (IL-8) exacerbates cardiac injury by enhancing leukocyte activation and accumulation [128,129]. This inflammatory state is compounded by oxidative stress linked to a decrease in antioxidant defenses and an increase in free radicals, with reperfusion injury being the main source of ROS. After a period of ischemia where there is a decrease in ATP production, causing changes in Na, Ca, and intracellular pH, the restoration of perfusion causes an explosive increase in ROS that generates significant cellular damage [130]. In this context, some findings regarding molecular markers supporting this process are the preoperative transcriptome analysis of atrial tissue from patients developing POAF, which revealed increased levels of tumor necrosis factor alpha, interleukin (IL) 6, and nuclear factor of light polypeptide kappa gene enhancer of B-cell mRNA (NF-κB), with decreased antioxidant defenses with lower mRNA levels of GSH synthetase, GSH reductase, and mitochondrial superoxide dismutase 2 (SOD2), as well as studies of pericardial fluid in patients developing POAF demonstrating increased IL-6 and myeloperoxidase (MPO) [131,132,133].

#### 4.2.3. Ischemic Stroke

This setting accounts for approximately 87% of all strokes [134]. It initiates with the blockage of cerebral arteries, diminishing blood flow to the brain. This reduction results in inadequate supplies of blood glucose and oxygen, triggering metabolic alterations, cellular demise, and damage to the brain [135].

In the case of ischemic stroke, there is also ischemia–reperfusion damage caused by an imbalance between free radicals and the antioxidant defense system. However, the brain consumes approximately 20% of the total blood flow with high oxygen demand, making it less tolerant to hypoxia and damage from reactive oxygen species (ROS) compared to the heart, with relatively lower antioxidant activity [136,137]. 

Neuronal function relies on ATP; thus, in this situation, the maintenance of the transmembrane gradient and neuronal signaling is disrupted. The activity of the Na/K ATPase pump is blocked, leading to increased intracellular calcium influx and elevated ROS production. Initially, during the hypoxia phase, ROS are primarily generated by mitochondria, while during reperfusion, they are more associated with increased activity of xanthine oxidase and NADPH oxidase [136,138].

Excessive reactive oxygen species (ROS) not only lead to cellular destruction, lipid peroxidation, and the oxidation of proteins, but also impact vascular tone, platelet activity, and endothelial permeability. This imbalance can facilitate leukocyte infiltration and edema and the accumulation of amyloid proteins in the brain, triggering a neurodegenerative response and neuronal dysfunction [139]. The mechanisms of neuronal death are diverse, such as apoptosis, necrosis, necroptosis, ferroptosis, and more [140].

## 5. Multitherapeutic Approaches Using Natural Antioxidants in Oxidative Stress-Related Diseases

### 5.1. Non-Alcoholic Fatty Liver Disease 

Weight loss is currently recognized as the most effective approach to treating NAFLD [106]. However, studies indicate that weight loss alone may not suffice. For instance, diets promoting rapid weight reduction with low carbohydrate or high fat content may prove inadequate. This suggests that the composition of the diet itself plays a crucial role, pointing towards a potential contribution of antioxidants [141].

Xie et al. found that elevated levels of vitamin C correlate with a reduced risk of NAFLD [142]. Similarly, a study showed that 12 weeks of vitamin C supplementation improved liver health and glucose metabolism in individuals with NAFLD [143]. Nobili et al. demonstrated that interventions involving dietary modifications and increased physical activity led to weight loss and notably improved liver histology and laboratory results in pediatric NAFLD patients. However, supplementation with alpha-tocopherol and ascorbic acid did not enhance the effectiveness of these lifestyle changes alone [144].

A randomized controlled trial (RCT) found no statistically significant difference in liver fat content among subjects supplemented with resveratrol, although it was deemed safe and well tolerated [145]. In another study, resveratrol showed improvements in various parameters in NAFLD patients, including AST, glucose, and LDL cholesterol levels [146].

A meta-analysis revealed that curcumin reduced BMI, lowered ALT and AST levels, and decreased triglycerides without significant adverse effects. However, there was no significant difference in LDL-C compared to the placebo group. Conversely, resveratrol supplementation did not result in differences in BMI, ALT, AST, triglyceride, or LDL-C levels, nor did it cause significant adverse effects [147]. Yang et al. demonstrated a reduction in serum transaminase levels and an improvement in histological abnormalities with quercetin supplementation. Additionally, it restored superoxide dismutase, catalase, and glutathione levels [148].

Regarding astaxanthin, an RCT indicated significant improvements in oxidative stress markers and total antioxidant capacity, with promising results observed in rat studies [149,150]. Moreover, increased serum carotenoids were associated with NAFLD improvement [151]. Lycopene prevented NAFLD development in rats fed a high-fat and/or cholesterol diet [152]. Another study concluded that the combination of rosuvastatin and beta-carotene was more beneficial than rosuvastatin alone in rats with NAFLD [153].

Thus, dietary intervention stands as a cornerstone in preventing and managing NAFLD, offering a cost-effective, non-invasive, and low-risk strategy. In this regard, one dietary pattern supported by ample scientific evidence is the Mediterranean diet, which includes a diverse array of natural antioxidants and anti-inflammatory agents, particularly polyphenols, carotenoids, and vitamins. Robust adherence to this diet has demonstrated, in various clinical trials, a reduction in the incidence and progression of fatty liver disease compared to conventional low-fat diets [154] (Table 1).

### 5.2. Ischemia–Reperfusion Injury

#### 5.2.1. Acute Myocardial Infarction

After blood flow is restored, specific antioxidant enzymes decrease in activity, and markers of oxidative stress increase. Nevertheless, vitamin C supplementation has been observed to normalize or nearly normalize these levels and has shown promising clinical outcomes [155,156,157]. However, in the study of Ramos et al., the administration of vitamin C did not reduce the infarct size, which makes the results still not entirely satisfactory [118] (Table 1). 

Resveratrol has demonstrated the ability to decrease oxidative stress levels in both in vivo and in vitro experiments, improving cardiac function, decreasing infarct size, and enhancing the activity of antioxidant enzymes such as superoxide dismutase (SOD) and glutathione peroxidase (GSH-PX) in rats [158,159]. However, more clinical studies are needed. Quercetin preconditioning was found to enhance heart function, decrease MDA levels, and increase the activity of antioxidant enzymes in rats [160]. Nevertheless, similar to resveratrol, further studies are essential to comprehensively explore its impact. 

A curcumin analog reduced the size of the infarct and myocardial apoptosis [161]. A meta-analysis of preclinical studies in animals suggested that curcumin could be beneficial for myocardial ischemia–reperfusion injury, improving oxidative stress parameters, Astaxanthin has exhibited potential benefits in this same context in both in vitro and ex vivo models (Figure 3) [162,163,164].

#### 5.2.2. Postoperative Atrial Fibrillation

As mentioned previously, oxidative stress plays a pivotal role in the development of postoperative atrial fibrillation (POAF). In this context, the investigation of natural antioxidant therapies to prevent the occurrence of POAF has gained importance. These therapies have primarily focused on the use of polyunsaturated fatty acids (PUFAs) and vitamins.

Omega-3 PUFAs (n-3 PUFAs), such as eicosapentaenoic acid (EPA) and docosahexaenoic acid (DHA), which are naturally present in fish, have been among the antioxidant treatments proposed for preventing POAF. The potential of n-3 PUFAs as a therapy is grounded in their capacity, demonstrated primarily in animal models, to generate a controlled increase in oxidative stress due to their susceptibility to lipid peroxidation. This controlled oxidative stress amplifies and positively modulates the cell’s antioxidant defense system. Additionally, n-3 PUFAs can electrically stabilize cardiac cell membranes and, concurrently, regulate calcium (Ca+) currents [165,166].

Regarding clinical trials that have sought to establish clinical efficacy in preventing POAF, the results have shown significant heterogeneity and contradictions. Some studies dismiss the protective role of PUFAs due to a neutral or even adverse effect, while others successfully demonstrate the anti-arrhythmic benefits of PUFAs [167]. This diversity in outcomes can be attributed to variations in the underlying oxidative burden of each patient and the method of administration. All randomized placebo-controlled studies using a formulation containing PUFAs in a 1:24 EPA:DHA ratio failed to demonstrate a beneficial effect [168,169,170,171]. Conversely, trials performed with this ratio equal to 0.5 reported a beneficial effect in POAF prevention [172,173,174]. Moreover, a metaregression analysis showed a trend toward a benefit of an EPA:DHA ratio of 0.5 [175] (Table 1). 

Concerning the use of vitamins, the utilization of vitamin C and vitamin E stands out. Clinical trials supporting the use of vitamin C include a meta-analysis that reviewed 28 controlled clinical trials where vitamin C was employed to prevent POAF in cardiac surgery. This meta-analysis demonstrated a reduction in the incidence of POAF, as well as a decrease in the duration of ICU stays and overall hospitalization [176]. On the other hand, the use of vitamin E by itself has not been extensively investigated, as it has not shown the potential to reduce oxidative stress at the cardiovascular level. High doses of vitamin E may displace other fat-soluble antioxidants, increasing susceptibility to oxidative damage [177]. However, synergistic therapy involving vitamin C, vitamin E, and n-3 PUFAs has demonstrated the best potential benefit in preventing POAF, as evident in a meta-analysis showing a 68% reduction in the incidence of POAF compared to the control group [167] (Table 1).

Another pivotal study in this field emphasizes the role of diet as a factor that modifies our baseline antioxidant levels before surgery. For instance, a study evaluating the connection between adherence to a Mediterranean diet, rich in antioxidants, and the development of POAF, revealed that the prolonged consumption of antioxidant-rich foods is associated with a reduced incidence of postoperative atrial fibrillation in patients undergoing cardiac surgery [178]. This confirms that the consumption of multiple antioxidants with diverse properties can act synergistically in preventing POAF.

#### 5.2.3. Stroke

Lower plasma levels of vitamin C have been observed in patients with stroke; however, the administration of vitamin C post-stroke has not necessarily resulted in clinical improvement in those individuals [179,180]. In another study, a combination of aspirin and vitamin C was administered, resulting in elevated vitamin C levels and reduced lipid peroxidation compared to individuals treated solely with aspirin [181] (Table 1).

Resveratrol has demonstrated therapeutic potential in rats by decreasing oxidative stress markers and showing improvement in neurological function, as well as a decrease in infarct size in a study [182,183]. On the other hand, curcumin has also shown potential benefits in ischemic stroke in rats, reducing neurological deficit score and infarct size, increasing antioxidant enzymes, and decreasing markers of oxidative stress [184]. In a study in rats, quercetin demonstrated a reduction in infarct size and an upregulation of antioxidant status [185].

In a study, it was revealed that the plasma levels of various carotenoids tend to decrease immediately after an ischemic stroke [186]. In addition, a reduction in alpha- and beta-carotene levels has been observed in stroke patients compared to healthy individuals [187]. 

**Table 1:** Summary of clinical studies mentioned above.

Disease	Study Details	“*n*”	Main Findings	Reference
**NAFLD**	Vitamin C measurement in patients with NAFLD	4.494	Inverse association between serum VC levels and NAFLD	[142]
	12 weeks of oral treatment with low/medium/high doses of VC	84	VC supplementation, specially medium dose (1000 mg/day), improved liver health and glucose metabolism	[143]
	Lifestyle intervention with or without antioxidant therapy (alpha-tocopherol and vitamin C)	53	Antioxidant therapy plus lifestyle did not have better results than lifestyle alone in liver histology and laboratory abnormalities	[144]
	Resveratrol supplementation in overweight, obese, and insulin-resistant patients.	112	Resveratrol was well tolerated, but it did not significantly impact liver fat content and cardiometabolic risk	[145]
	Subjects with NAFLD were given resveratrol daily for 3 months versus a placebo	60	In the resveratrol group, there was a reduction in different parameters, showing beneficial effects in comparison to the placebo group	[146]
	Obese patients were given low (5 mg) versus high (20 mg) doses of astaxanthin	23	Astaxanthin supplementation was associated with an improvement in OS markers	[150]
	Baseline serum concentrations of carotenoids, followed by abdominal US at 3 and 6 years	2687	Higher serum carotenoid concentration was associated with NAFLD improvement	[151]
**Acute myocardial infarction**	Effect of intravenous and intracoronary vitamin C in patients undergoing PCI	252	Patients with VC had significantly lower troponin T and CK MB levels at 12 and 6 h	[155]
	VC administration prior to PCI followed by oral VC + vitamin E for 84 days	53	Left ventricular ejection fraction was significantly higher in the high ascorbate group than in the low ascorbate group	[156]
	VC in patients after thrombolysis in AMI for 5 days	65	OS markers were restored almost back to normal values after VC administration	[157]
**POAF**	Meta-analysis of PUFA and vitamin C/E in the incidence of POAF	3137	PUFA alone did not reduce the incidence of POAF, but PUFA plus vitamin C and E had a significant effect in preventing POAF	[167]
	Perioperative addition of PUFAs in patients scheduled for cardiac surgery	1516	The risk of developing POAF was not reduced with the addition of PUFAs	[168]
	Patients undergoing coronary artery bypass graft surgery treated with oral PUFAs before surgery	260	Oral supplementation of PUFAs before surgery did not reduce the risk of POAF	[169]
	Patients with cardiac surgery were given PUFAs 5–7 days before the procedure and after until hospital discharge	168	There was not beneficial effect in the incidence of POAF in patients supplemented with PUFAs	[170]
	Patients undergoing cardiac surgery were given PUFAs for at least 5 days before	108	Omega-3 PUFA did not reduce the risk of AF after coronary artery bypass graft surgery	[171]
	Preoperative PUFA therapy in patients undergoing cardiac surgery	530	Preoperative PUFA therapy is associated with a decreased incidence of early AF after cardiac surgery but not late AF	[172]
	Pre- and postoperative treatment with PUFAs at least 5 days before elective cardiac surgery	160	PUFA administration substantially reduced the incidence of POAF (54.4%) and was associated with a shorter hospital stay	[173]
	Pre- and postoperative administration of PUFAs in patients undergoing elective cardiac surgery	201	There was a significant reduction in POAF in patients treated with PUFAs	[174]
	Long-term intake of antioxidant-rich foods in patients undergoing cardiac surgery	217	Long-term consumption of antioxidant-rich foods was associated with a reduced risk of developing POAF	[178]
**Stroke**	Nutritional status and plasma levels of vitamin C and E in patients 2–5 days after stroke onset	15	Stroke patients had significantly lower plasma levels of vitamin C and higher oxidative stress markers	[179]
	Vitamin C therapy for 10 days after stroke onset	60	Vitamin C elevated serum antioxidant levels, but it did not improve the clinical and functional status of the patient after 3 months	[180]
	Vitamin C plus aspirin vs. aspirin alone in patients after ischemic stroke	59	Vitamin C plus aspirin significantly decreases lipid peroxidation	[181]
	Over-time changes in a number of carotenoids during the first hours after the occurrence of ischemic stroke	28	The majority of plasma carotenoids are lowered immediately after an ischemic stroke	[186]
	Plasma levels of lipophilic antioxidant vitamins and neurological deficits after 48 h of stroke onset	68	Plasma levels of alpha- and beta-carotene were lower in patients with stroke. There was a negative association between neurologic deficit and plasma levels of carotenoids	[187]

## 6. In Silico Studies on Natural Antioxidants

In silico studies on antioxidants are now a crucial aspect of modern research, providing insights into the molecular mechanisms and potential therapeutic applications of these compounds. These studies use computational methods to simulate and analyze the interactions between antioxidants and biological targets.

One key area is molecular docking and dynamic simulations, which predict the binding affinity and interaction of antioxidants with specific target proteins, such as enzymes involved in oxidative stress pathways [188]. For instance, studies have investigated phytochemicals and natural compounds with antioxidant activity interacting with enzymes like NADPH oxidase and superoxide dismutase [189,190,191].

Another important approach is quantitative structure–activity relationship (QSAR) analysis, which relates the chemical structure of antioxidants to their biological activity. These models help predict the antioxidant activity of new compounds based on their molecular descriptors, such as the QSAR analysis of flavonoids [192].

Moreover, molecular dynamics (MD) simulations provide insights into the stability and dynamics of antioxidant–protein complexes over time. These simulations help us understand the conformational changes and binding stability of antioxidants. For example, MD simulations have been used to study vitamin E interacting with lipid membranes, revealing its protective effects against lipid peroxidation [193,194].

Furthermore, a virtual screening study by Lavecchia and Giovanni identified several potential antioxidant compounds from a library of natural products. The top hits were further validated using docking and MD simulations, highlighting the effectiveness of virtual screening in discovering new antioxidants [195]. These data can also contribute to pharmacophore modeling, which helps in designing new compounds by identifying the essential features required for antioxidant activity [196] 

Computational approaches are powerful and versatile tools in modern scientific research for understanding and manipulating biological and chemical systems. They complement traditional experimental methods by providing valuable insights and accelerating the pace of discovery and development across various fields. This is particularly useful in the clinical setting for developing treatment strategies for prevalent diseases. However, it is important to note that in silico studies have limitations. Virtual models of biological systems inherently possess a margin of error, as they are based on digital representations rather than real products [197]. Consequently, in silico studies are not intended to replace the classical approach of molecular discovery and design, but to complement it. Another drawback is that only a small percentage of drugs developed through these methods demonstrate efficacy in clinical settings, a situation exacerbated by the lack of standardized validation [198,199]. To enhance confidence in these models, it has been proposed to combine conventional clinical trials with modeling tools, comparing the predictions of computational models with clinical decisions [199]. This strategy can build stronger evidence, offsetting the limitations of each method and improving the understanding of computational modeling.

## 7. Concluding Remarks and Future Perspectives

Current knowledge about natural antioxidants and their mechanisms of action remains incomplete. Consequently, many clinical studies have not yielded satisfactory results, hindering the development of alternative therapies for diseases where oxidative stress is a key factor in their pathogenesis. However, this review shows that in specific contexts, antioxidants used in a reasoned manner based on their properties can be beneficial. Nonetheless, the hypothesis that combining these compounds as part of a multitherapeutic approach yields better outcomes has not been fully validated due to limited evidence in this area. Future studies are expected to explore the potential synergistic effects of these compounds.

In the case of non-alcoholic fatty liver disease (NAFLD), although weight loss remains the most effective treatment, the inclusion of antioxidants in the diet plays a crucial role. Adding vitamins such as C and E; polyphenols such as resveratrol, curcumin, and quercetin; and carotenoids like astaxanthin and lycopene holds significant potential for improving liver health and metabolic parameters.

Furthermore, natural antioxidants have shown promise in the context of ischemia–reperfusion, particularly in acute myocardial infarction and postoperative atrial fibrillation (POAF). Vitamin C, resveratrol, and quercetin have been effective in reducing oxidative stress markers and improving cardiac function in animal models and clinical studies of acute myocardial infarction. Additionally, combining vitamins C and E with PUFAs appears to offer consistent benefits in preventing POAF, suggesting that a synergistic approach may be more effective for this condition.

Regarding stroke studies, although lower plasma levels of antioxidants such as vitamin C and carotenoids are associated with stroke patients, supplementation has not consistently resulted in clinical improvement. Preclinical studies with resveratrol, curcumin, and quercetin show potential benefits, but these results have yet to be confirmed in clinical trials with patients.

A crucial aspect of using natural antioxidants is their integration into the daily diet, which could be fundamental for preventing various diseases, especially cardiovascular and metabolic diseases where oxidative stress plays an essential role. Promoting dietary patterns that incorporate a wide range of natural antioxidants offers a cost-effective and low-risk intervention strategy with potential health benefits, with the Mediterranean diet being one of the most studied and supported by evidence.

Finally, an important development is the emergence of in silico studies, which use computational methods to understand the molecular interactions and stability of antioxidant compounds. This technology will enhance our understanding of the properties of each natural antioxidant, facilitating the creation of specific therapies for various pathologies in the future. 

## Authors Contributions

V.P.-G., C.R.-S., F.G.-H., T.G.-F. and R.R. contributed to the elaboration of the idea and structure of the manuscript. F.G.-H., T.G.-F., V.P.-G. and A.C.-C. wrote the manuscript, and R.R. helped with the editing. C.R.-S., L.S., S.C. and R.R. supervised the review and contributed changes. T.G.-F. and F.G.-H. prepared the figures. All authors have read and agreed to the published version of the manuscript.

## Data Availability

No new data were created or analyzed in this study. Data sharing is not applicable to this article.
